# Reconstruction of Severe Nasal Septum Deformity

**DOI:** 10.29252/wjps.7.3.385

**Published:** 2018-09

**Authors:** Yakup Cil

**Affiliations:** Department of Plastic Surgery, Ataturk Training and Research Hospital, Ankara, Turkey

**Keywords:** Reconstruction, Nasal septum, Deformity

**DEAR EDITOR**

Different surgical techniques have been described for treatment of severe nasal septum deformities.^1^^-^^4^ We used iliac bone with costal cartilage graft for Tessier No O nose deformity. A 22-year-old male patient admitted to our clinic due to severe functional and aesthetic nasal problems (Figure 1). Physical examination showed that the nasal septum was severely distorted. Nasal structures were exposed through an open rhinoplasty approach under patient general anesthesia. Septal cartilage sculpturing was not possible and distorted cartilage septum remnant was removed. Rigid iliac bone graft and costal cartilage was taken and prepared like as sandwich for nasal septum reconstruction (Figure 2). 

**Fig. 1:**
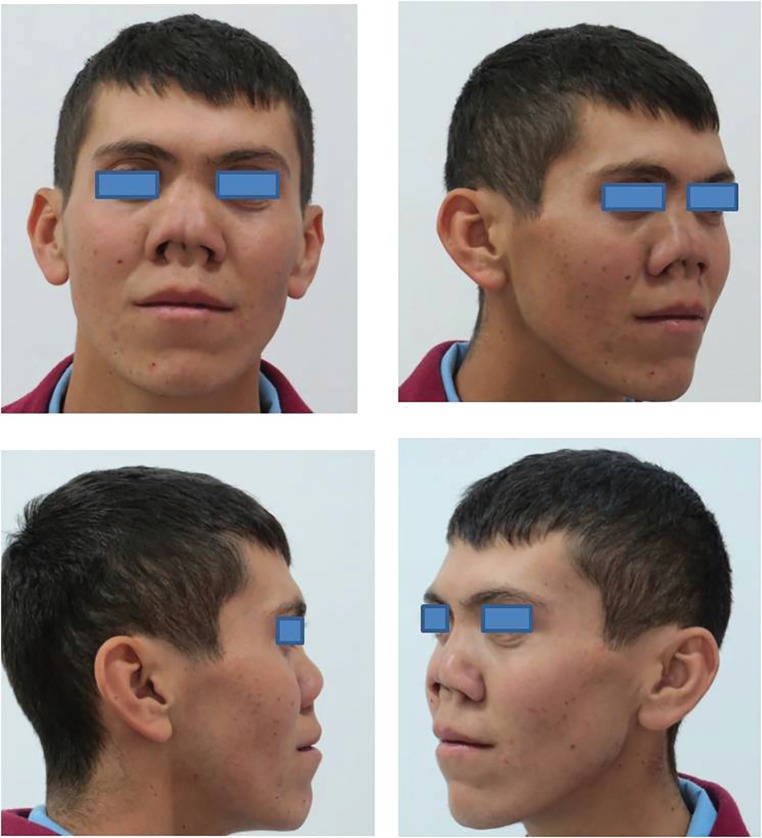
Preoperative views of the patient with Tessier No 0 nose deformity

**Fig. 2:**
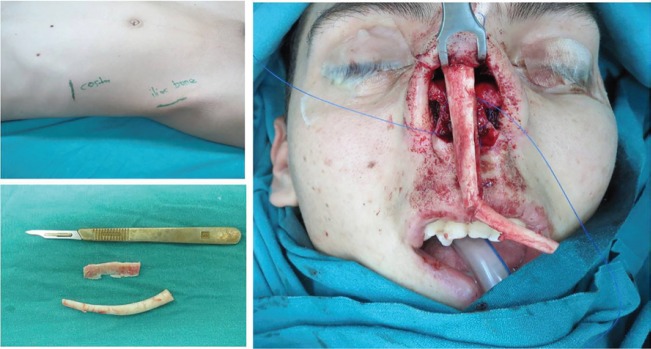
İliac bone and costal cartilage taken site views. İliac bone was inset in costal cartilage as sandwich before nasal placement

Duration of the operation was three hours. After closure, nasal packing was applied. The packing and the nasal splint were removed at 3th and 7th days, respectively. Patient has recovered without problems. No donor site complication has been observed. Aesthetic and functional results were acceptable (Figure 3). Different techniques have been described in the literature for severe nose deformity corrections.^1^^-^^8^ In the management of this case; iliac bone graft with costal cartilage has been used for reconstruction of severe nasal septum deformity. The bone and cartilage combination may be used in selected nose deformity cases.

**Fig. 3:**
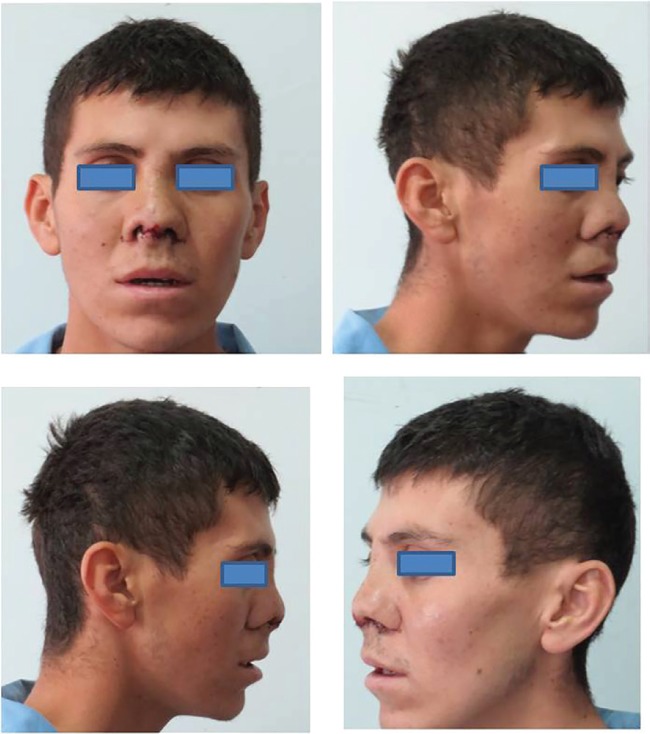
Postoperative views of the nose

## CONFLICT OF INTEREST

The authors declare no conflict of interest.
